# 

**Published:** 1886-03

**Authors:** 


### CONTENTS:

Art. I—Odonto-Chirurgical Society.................................... 480

Art. II—Non-Fistulous Abscess. By Dr. Kulp.................................... 491

Art. III—Odontological Society of Great Britain.................................... 493

Art. IV—Atmospheric Anæsthesia. By W. H. Richards, D. D. S.................................... 502

Art. V—Dental Spiritualism. By Louis Ottofy, D. D. S.................................... 504

Art. VI—The American Dental Association.................................... 514

Art. VII—Gas Furnaces vs. Coal or Coke. By H. C. Land.................................... 518

Chicago Dental Society.................................... 520

Illinois State Dental Society.................................... 521

### EDITORIAL, ETC.

Annual Commencement of the Dental Department of the University of Maryland.... 521
The International Medical Congress.................................... 524

### MONTHLY SUMMARY.

Capsicum Bags.................................... 526

To Mend a Broken File.................................... 527

Glycerine in Dryness of the Tongue.................................... 528

Preservation of Cocaine Solutions.................................... 528

